# Supplementary material for: Self-cyclisation as a general and efficient platform for peptide and protein macrocyclisation
Source: Commun Chem. 2023 Mar 4;6:48. doi: 10.1038/s42004-023-00841-5 (PMC9985607; doi:10.1038/s42004-023-00841-5)
Supplement: Supplementary file 2 — Description of Additional Supplementary Files [file 42004_2023_841_MOESM2_ESM.pdf]

Description of Additional Supplementary Files

File name: Supplementary Data 1

Description: Complete sequences of all autocyclases generated forthis work.

File name: Supplementary Data 2

Description: Initial and final configurations (PDB format) for the final molecular dynamics simulation run of each system described.

File name: Supplementary Data 3

Description: Mass spectrometry data used to characterize cyclisation kinetics.

File name: Supplementary Data 4

Description: Imaging data used to characterize nanodisc biodistribution.
