# Supplementary material for: Self-cyclisation as a general and efficient platform for peptide and protein macrocyclisation
Source: Commun Chem. 2023 Mar 4;6:48. doi: 10.1038/s42004-023-00841-5 (PMC9985607; doi:10.1038/s42004-023-00841-5)
Supplement: Supplementary file 3 — Supplementary Data 1 [file 42004_2023_841_MOESM3_ESM.pdf]

Supplementary Data 1

1. *a*MSP6-L_{12} (MSP6-LPGTG-L12-SrtA-His_{10})

```
atggcttcgtccgagaatttgtacttccaaggatcgacgttttccaagttacgcgaacag
 M  A  S  S  E  N  L  Y  F  Q  G  S  T  F  S  K  L  R  E  Q
ttaggacccgtaacgcaggaattctgggacaaccttgagaaagagacggaaggccttcgc
 L  G  P  V  T  Q  E  F  W  D  N  L  E  K  E  T  E  G  L  R
caggagatgtcaaaagaccttgaggaagtgaaggctaaggtacaaccctatagtgatgag
 Q  E  M  S  K  D  L  E  E  V  K  A  K  V  Q  P  Y  S  D  E
ttgcgccagcgtttggccgcacgtttagaggctttgaaagagaatggcggtgcccgtctg
 L  R  Q  R  L  A  A  R  L  E  A  L  K  E  N  G  G  A  R  L
gccgagtaccatgcaaaggcgacagaacatttgtccaccttgagcgagaaagctaaaccg
 A  E  Y  H  A  K  A  T  E  H  L  S  T  L  S  E  K  A  K  P
gctctggaggacttgcgtcagggcttgcttccggtacttgaatcattcaaggtgtccttt
 A  L  E  D  L  R  Q  G  L  L  P  V  L  E  S  F  K  V  S  F
ctgtctgccttagaagagtatactaagaagcttaacacacaactgcctggcacaggtgct
 L  S  A  L  E  E  Y  T  K  K  L  N  T  Q  L  P  G  T  G  A
gcagctttagagggtaccctggtgccgcgcagccaagctaaacctcaaattccgaaagat
 A  A  L  E  G  T  L  V  P  R  S  Q  A  K  P  Q  I  P  K  D
aaatcgaaagtggcaggctatattgaaattccagatgctgatattaaagaaccagtatat
 K  S  K  V  A  G  Y  I  E  I  P  D  A  D  I  K  E  P  V  Y
ccaggaccagcaacacctgaacaattaaatagaggtgtaagctttgcagaagaaaatgaa
 P  G  P  A  T  P  E  Q  L  N  R  G  V  S  F  A  E  E  N  E
tcactagatgatcaaaatatttcaattgcaggacacactttcattgaccgtccgaactat
 S  L  D  D  Q  N  I  S  I  A  G  H  T  F  I  D  R  P  N  Y
caatttacaaatcttaaagcagccaaaaaaggtagtatggtgtactttaaagttggtaat
 Q  F  T  N  L  K  A  A  K  K  G  S  M  V  Y  F  K  V  G  N
gaaacacgtaagtataaaatgacaagtataagagatgttaagcctacagatgtaggagtt
 E  T  R  K  Y  K  M  T  S  I  R  D  V  K  P  T  D  V  G  V
ctagatgaacaaaaaggtaaagataaacaattaacattaattacttgtgatgattacaat
 L  D  E  Q  K  G  K  D  K  Q  L  T  L  I  T  C  D  D  Y  N
gaaaagacaggcgtttgggaaaaacgtaaaatctttgtagctacagaagtcaaactcgag
 E  K  T  G  V  W  E  K  R  K  I  F  V  A  T  E  V  K  L  E
caccaccaccaccaccaccatcatcatcattga
 H  H  H  H  H  H  H  H  H  H  -
```

2. *a*MSP6-L$_{14D}$ (MSP6-LPGTG-L14D-SrtA-His$_{10}$)

```
atggcttcgtccgagaatttgtacttccaaggatcgacgttttccaagttacgcgaacag
 M  A  S  S  E  N  L  Y  F  Q  G  S  T  F  S  K  L  R  E  Q
ttaggacccgtaacgcaggaattctgggacaaccttgagaaagagacggaaggccttcgc
 L  G  P  V  T  Q  E  F  W  D  N  L  E  K  E  T  E  G  L  R
caggagatgtcaaaagaccttgaggaagtgaaggctaaggtacaaccctatagtgatgag
 Q  E  M  S  K  D  L  E  E  V  K  A  K  V  Q  P  Y  S  D  E
ttgcgccagcgtttggccgcacgtttagaggctttgaaagagaatggcggtgcccgtctg
 L  R  Q  R  L  A  A  R  L  E  A  L  K  E  N  G  G  A  R  L
gccgagtaccatgcaaaggcgacagaacatttgtccaccttgagcgagaaagctaaaccg
 A  E  Y  H  A  K  A  T  E  H  L  S  T  L  S  E  K  A  K  P
gctctggaggacttgcgtcagggcttgcttccggtacttgaatcattcaaggtgtccttt
 A  L  E  D  L  R  Q  G  L  L  P  V  L  E  S  F  K  V  S  F
ctgtctgccttagaagagtatactaagaagcttaacacacaactgcctggtaccggggga
 L  S  A  L  E  E  Y  T  K  K  L  N  T  Q  L  P  G  T  G  G
tcgggaggttcaggtgggtccggtggtagtggtgggagtcaagctaaacctcaaattccg
 S  G  G  S  G  G  S  G  G  S  G  G  S  Q  A  K  P  Q  I  P
aaagataaatcgaaagtggcaggctatattgaaattccagatgctgatattaaagaacca
 K  D  K  S  K  V  A  G  Y  I  E  I  P  D  A  D  I  K  E  P
gtatatccaggaccagcaacacctgaacaattaaatagaggtgtaagctttgcagaagaa
 V  Y  P  G  P  A  T  P  E  Q  L  N  R  G  V  S  F  A  E  E
aatgaatcactagatgatcaaaatatttcaattgcaggacacactttcattgaccgtccg
 N  E  S  L  D  D  Q  N  I  S  I  A  G  H  T  F  I  D  R  P
aactatcaatttacaaatcttaaagcagccaaaaaaggtagtatggtgtactttaaagtt
 N  Y  Q  F  T  N  L  K  A  A  K  K  G  S  M  V  Y  F  K  V
ggtaatgaaacacgtaagtataaaatgacaagtataagagatgttaagcctacagatgta
 G  N  E  T  R  K  Y  K  M  T  S  I  R  D  V  K  P  T  D  V
ggagttctagatgaacaaaaaggtaaagataaacaattaacattaattacttgtgatgat
 G  V  L  D  E  Q  K  G  K  D  K  Q  L  T  L  I  T  C  D  D
tacaatgaaaagacaggcgtttgggaaaaacgtaaaatctttgtagctacagaagtcaaa
 Y  N  E  K  T  G  V  W  E  K  R  K  I  F  V  A  T  E  V  K
ctcgagcaccaccaccaccaccaccatcatcatcattga
 L  E  H  H  H  H  H  H  H  H  H  H  -
```

3. *a*MSP7-L_{12} (MSP7-LPGTG-L12-SrtA-His_{10})

```
atggcttcgtccgagaatttgtacttccaaggatcgacgttttccaagttacgcgaacag
 M  A  S  S  E  N  L  Y  F  Q  G  S  T  F  S  K  L  R  E  Q
ttaggacccgtaacgcaggaattctgggacaaccttgagaaagagacggaaggccttcgc
 L  G  P  V  T  Q  E  F  W  D  N  L  E  K  E  T  E  G  L  R
caggagatgtcaaaagaccttgaggaagtgaaggctaaggtacaacccttgggggaggag
 Q  E  M  S  K  D  L  E  E  V  K  A  K  V  Q  P  L  G  E  E
atgcgtgatcgcgcccgcgcccacgtggatgcattgcgcacgcatttagctccatatagt
 M  R  D  R  A  R  A  H  V  D  A  L  R  T  H  L  A  P  Y  S
gatgagttgcgccagcgtttggccgcacgtttagaggctttgaaagagaatggcggtgcc
 D  E  L  R  Q  R  L  A  A  R  L  E  A  L  K  E  N  G  G  A
cgtctggccgagtaccatgcaaaggcgacagaacatttgtccaccttgagcgagaaagct
 R  L  A  E  Y  H  A  K  A  T  E  H  L  S  T  L  S  E  K  A
aaaccggctctggaggacttgcgtcagggcttgcttccggtacttgaatcattcaaggtg
 K  P  A  L  E  D  L  R  Q  G  L  L  P  V  L  E  S  F  K  V
tcctttctgtctgccttagaagagtatactaagaagcttaacacacaactgcctggcaca
 S  F  L  S  A  L  E  E  Y  T  K  K  L  N  T  Q  L  P  G  T
ggtgctgcagctttagagggtaccctggtgccgcgcagccaagctaaacctcaaattccg
 G  A  A  A  L  E  G  T  L  V  P  R  S  Q  A  K  P  Q  I  P
aaagataaatcgaaagtggcaggctatattgaaattccagatgctgatattaaagaacca
 K  D  K  S  K  V  A  G  Y  I  E  I  P  D  A  D  I  K  E  P
gtatatccaggaccagcaacacctgaacaattaaatagaggtgtaagctttgcagaagaa
 V  Y  P  G  P  A  T  P  E  Q  L  N  R  G  V  S  F  A  E  E
aatgaatcactagatgatcaaaatatttcaattgcaggacacactttcattgaccgtccg
 N  E  S  L  D  D  Q  N  I  S  I  A  G  H  T  F  I  D  R  P
aactatcaatttacaaatcttaaagcagccaaaaaaggtagtatggtgtactttaaagtt
 N  Y  Q  F  T  N  L  K  A  A  K  K  G  S  M  V  Y  F  K  V
ggtaatgaaacacgtaagtataaaatgacaagtataagagatgttaagcctacagatgta
 G  N  E  T  R  K  Y  K  M  T  S  I  R  D  V  K  P  T  D  V
ggagttctagatgaacaaaaaggtaaagataaacaattaacattaattacttgtgatgat
 G  V  L  D  E  Q  K  G  K  D  K  Q  L  T  L  I  T  C  D  D
tacaatgaaaagacaggcgtttgggaaaaacgtaaaatctttgtagctacagaagtcaaa
 Y  N  E  K  T  G  V  W  E  K  R  K  I  F  V  A  T  E  V  K
ctcgagcaccaccaccaccaccaccatcatcatcattga
 L  E  H  H  H  H  H  H  H  H  H  H  -
```

*4. a*MSP7-L_{14D} (MSP7-LPGTG-L14D-SrtA-His_{10})

```
atggcttcgtccgagaatttgtacttccaaggatcgacgttttccaagttacgcgaacag
 M   A   S   S   E   N   L   Y   F   Q   G   S   T   F   S   K   L   R   E   Q
ttaggacccgtaacgcaggaattctgggacaaccttgagaaagagacggaaggccttcgc
 L   G   P   V   T   Q   E   F   W   D   N   L   E   K   E   T   E   G   L   R
caggagatgtcaaaagaccttgaggaagtgaaggctaaggtacaacccttggggaggag
 Q   E   M   S   K   D   L   E   E   V   K   A   K   V   Q   P   L   G   E   E
atgcgtgatcgcgcccgcgcccacgtggatgcattgcgcacgcatttagctccatatagt
 M   R   D   R   A   R   A   H   V   D   A   L   R   T   H   L   A   P   Y   S
gatgagttgcgccagcgtttggccgcacgtttagaggctttgaaagagaatggcggtgcc
 D   E   L   R   Q   R   L   A   A   R   L   E   A   L   K   E   N   G   G   A
cgtctggccgagtaccatgcaaaggcgacagaacatttgtccaccttgagcgagaaagct
 R   L   A   E   Y   H   A   K   A   T   E   H   L   S   T   L   S   E   K   A
aaaccggctctggaggacttgcgtcagggcttgcttccggtacttgaatcattcaaggtg
 K   P   A   L   E   D   L   R   Q   G   L   L   P   V   L   E   S   F   K   V
tcctttctgtctgccttagaagagtatactaagaagcttaacacacaactgcctggtacc
 S   F   L   S   A   L   E   E   Y   T   K   K   L   N   T   Q   L   P   G   T
ggggatcgggaggttcaggtgggtccggtggtagtggtgggagtcaagctaaacctcaa
 G   G   S   G   G   S   G   G   S   G   G   S   G   G   S   Q   A   K   P   Q
attccgaaagataaatcgaaagtggcaggctatattgaaattccagatgctgatattaaa
 I   P   K   D   K   S   K   V   A   G   Y   I   E   I   P   D   A   D   I   K
gaaccagtatatccaggaccagcaacacctgaacaattaaatagaggtgtaagctttgca
 E   P   V   Y   P   G   P   A   T   P   E   Q   L   N   R   G   V   S   F   A
gaagaaaatgaatcactagatgatcaaaatatttcaattgcaggacacactttcattgac
 E   E   N   E   S   L   D   D   Q   N   I   S   I   A   G   H   T   F   I   D
cgtccgaactatcaatttacaaatcttaaagcagccaaaaaaggtagtatggtgtacttt
 R   P   N   Y   Q   F   T   N   L   K   A   A   K   K   G   S   M   V   Y   F
aaagttggtaatgaaacacgtaagtataaaatgacaagtataagagatgttaagcctaca
 K   V   G   N   E   T   R   K   Y   K   M   T   S   I   R   D   V   K   P   T
gatgtaggagttctagatgaacaaaaaggtaaagataaacaattaacattaattacttgt
 D   V   G   V   L   D   E   Q   K   G   K   D   K   Q   L   T   L   I   T   C
gatgattacaatgaaaagacaggcgtttgggaaaaacgtaaaatctttgtagctacagaa
 D   D   Y   N   E   K   T   G   V   W   E   K   R   K   I   F   V   A   T   E
gtcaaactcgagcaccaccaccaccaccaccatcatcatcattga
 V   K   L   E   H   H   H   H   H   H   H   H   H   H   -
```

*5. a*MSP9-L_7-eSrtA (MSP9-LPGTG-L7-eSrtA-His_{10})

```
atggcttcgtccgagaatttgtacttccaaggatcgacgttttccaagttacgcgaacag
 M  A  S  S  E  N  L  Y  F  Q  G  S  T  F  S  K  L  R  E  Q
ttaggacccgtaacgcaggaattctgggacaaccttgagaaagagacggaaggccttcgc
 L  G  P  V  T  Q  E  F  W  D  N  L  E  K  E  T  E  G  L  R
caggagatgtcaaaagaccttgaggaagtgaaggctaaggtacaaccctatctggacgat
 Q  E  M  S  K  D  L  E  E  V  K  A  K  V  Q  P  Y  L  D  D
tttcaaaagaagtggcaagaagaaatggagttgtatcgtcaaaagttgaacctttgggg
 F  Q  K  K  W  Q  E  E  M  E  L  Y  R  Q  K  V  E  P  L  G
gaggagatgcgtgatcgcgcccgcgcccacgtggatgcattgcgcacgcatttagctcca
 E  E  M  R  D  R  A  R  A  H  V  D  A  L  R  T  H  L  A  P
tatagtgatgagttgcgccagcgtttggccgcacgtttagaggctttgaaagagaatggc
 Y  S  D  E  L  R  Q  R  L  A  A  R  L  E  A  L  K  E  N  G
ggtgcccgtctggccgagtaccatgcaaaggcgacagaacatttgtccaccttgagcgag
 G  A  R  L  A  E  Y  H  A  K  A  T  E  H  L  S  T  L  S  E
aaagctaaaccggctctggaggacttgcgtcagggcttgcttccggtacttgaatcattc
 K  A  K  P  A  L  E  D  L  R  Q  G  L  L  P  V  L  E  S  F
aaggtgtcctttctgtctgccttagaagagtatactaagaagcttaacacacaactgcct
 K  V  S  F  L  S  A  L  E  E  Y  T  K  K  L  N  T  Q  L  P
ggcacaggtgctgcagctttagagggtacccaagctaaaccgcagatccccaaagacaaa
 G  T  G  A  A  A  L  E  G  T  Q  A  K  P  Q  I  P  K  D  K
tctaaagttgcaggttatattgagatcccagacgcggatattaaggagcccgtgtatccg
 S  K  V  A  G  Y  I  E  I  P  D  A  D  I  K  E  P  V  Y  P
ggtcccgccactcgcgagcagttgaatcgcggagtctcctttgcagaggaaaatgaatcg
 G  P  A  T  R  E  Q  L  N  R  G  V  S  F  A  E  E  N  E  S
ttggatgaccagaatatctctattgccggtcatacattcatcgaccgtccaaattaccaa
 L  D  D  Q  N  I  S  I  A  G  H  T  F  I  D  R  P  N  Y  Q
ttcactaaccttaaagccgcgaaaaaggggtcgatggtctatttcaaggtgggcaatgaa
 F  T  N  L  K  A  A  K  K  G  S  M  V  Y  F  K  V  G  N  E
acacgcaaatataaaatgacttcgattcgtaacgtcaaaccaacggctgtggaagtgtta
 T  R  K  Y  K  M  T  S  I  R  N  V  K  P  T  A  V  E  V  L
gacgagcaaaaaggcaaggataagcaacttactttaattacgtgtgacgattataatgaa
 D  E  Q  K  G  K  D  K  Q  L  T  L  I  T  C  D  D  Y  N  E
gagacaggagtatgggagacacgcaaaatcttcgtggcgacggaggttaagctcgagcac
 E  T  G  V  W  E  T  R  K  I  F  V  A  T  E  V  K  L  E  H
caccaccaccaccaccatcatcatcattga
 H  H  H  H  H  H  H  H  H  -
```

*6. a*MSP9-L_7 (MSP9-LPGTG-L7-SrtA-His_{10})

```
atggcttcgtccgagaatttgtacttccaaggatcgacgttttccaagttacgcgaacag
 M  A  S  S  E  N  L  Y  F  Q  G  S  T  F  S  K  L  R  E  Q
ttaggacccgtaacgcaggaattctgggacaaccttgagaaagagacggaaggccttcgc
 L  G  P  V  T  Q  E  F  W  D  N  L  E  K  E  T  E  G  L  R
caggagatgtcaaaagaccttgaggaagtgaaggctaaggtacaaccctatctggacgat
 Q  E  M  S  K  D  L  E  E  V  K  A  K  V  Q  P  Y  L  D  D
tttcaaaagaagtggcaagaagaaatggagttgtatcgtcaaaaagttgaacctttgggg
 F  Q  K  K  W  Q  E  E  M  E  L  Y  R  Q  K  V  E  P  L  G
gaggagatgcgtgatcgcgcccgcgcccacgtggatgcattgcgcacgcatttagctcca
 E  E  M  R  D  R  A  R  A  H  V  D  A  L  R  T  H  L  A  P
tatagtgatgagttgcgccagcgtttggccgcacgtttagaggctttgaaagagaatggc
 Y  S  D  E  L  R  Q  R  L  A  A  R  L  E  A  L  K  E  N  G
ggtgcccgtctggccgagtaccatgcaaaggcgacagaacatttgtccaccttgagcgag
 G  A  R  L  A  E  Y  H  A  K  A  T  E  H  L  S  T  L  S  E
aaagctaaaccggctctggaggacttgcgtcagggcttgcttccggtacttgaatcattc
 K  A  K  P  A  L  E  D  L  R  Q  G  L  L  P  V  L  E  S  F
aaggtgtcctttctgtctgccttagaagagtatactaagaagcttaacacacaactgcct
 K  V  S  F  L  S  A  L  E  E  Y  T  K  K  L  N  T  Q  L  P
ggcacaggtgctgcagctttagagggtacccaagctaaacctcaaattccgaaagataaa
 G  T  G  A  A  A  L  E  G  T  Q  A  K  P  Q  I  P  K  D  K
tcgaaagtggcaggctatattgaaattccagatgctgatattaaagaaccagtatatcca
 S  K  V  A  G  Y  I  E  I  P  D  A  D  I  K  E  P  V  Y  P
ggaccagcaacacctgaacaattaaatagaggtgtaagctttgcagaagaaaatgaatca
 G  P  A  T  P  E  Q  L  N  R  G  V  S  F  A  E  E  N  E  S
ctagatgatcaaaatatttcaattgcaggacacactttcattgaccgtccgaactatcaa
 L  D  D  Q  N  I  S  I  A  G  H  T  F  I  D  R  P  N  Y  Q
tttacaaatcttaaagcagccaaaaaaggtagtatggtgtactttaaagttggtaatgaa
 F  T  N  L  K  A  A  K  K  G  S  M  V  Y  F  K  V  G  N  E
acacgtaagtataaaatgacaagtataagagatgttaagcctacagatgtaggagttcta
 T  R  K  Y  K  M  T  S  I  R  D  V  K  P  T  D  V  G  V  L
gatgaacaaaaaggtaaagataaacaattaacattaattacttgtgatgattacaatgaa
 D  E  Q  K  G  K  D  K  Q  L  T  L  I  T  C  D  D  Y  N  E
aagacaggcgtttgggaaaaacgtaaaatctttgtagctacagaagtcaaactcgagcac
 K  T  G  V  W  E  K  R  K  I  F  V  A  T  E  V  K  L  E  H
caccaccaccaccaccatcatcatcattga
 H  H  H  H  H  H  H  H  H  -
```

*7. a*MSP9-L$_{7D}$ (MSP9-LPGTG-L7D-SrtA-His$_{10}$)

```
atggcttcgtccgagaatttgtacttccaaggatcgacgttttccaagttacgcgaacag
 M  A  S  S  E  N  L  Y  F  Q  G  S  T  F  S  K  L  R  E  Q 
ttaggacccgtaacgcaggaattctgggacaaccttgagaaagagacggaaggccttcgc
 L  G  P  V  T  Q  E  F  W  D  N  L  E  K  E  T  E  G  L  R 
caggagatgtcaaaagaccttgaggaagtgaaggctaaggtacaaccctatctggacgat
 Q  E  M  S  K  D  L  E  E  V  K  A  K  V  Q  P  Y  L  D  D 
tttcaaaagaagtggcaagaagaaatggagttgtatcgtcaaaaagttgaacctttgggg
 F  Q  K  K  W  Q  E  E  M  E  L  Y  R  Q  K  V  E  P  L  G 
gaggagatgcgtgatcgcgcccgcgcccacgtggatgcattgcgcacgcatttagctcca
 E  E  M  R  D  R  A  R  A  H  V  D  A  L  R  T  H  L  A  P 
tatagtgatgagttgcgccagcgtttggccgcacgtttagaggctttgaaagagaatggc
 Y  S  D  E  L  R  Q  R  L  A  A  R  L  E  A  L  K  E  N  G 
ggtgcccgtctggccgagtaccatgcaaaggcgacagaacatttgtccaccttgagcgag
 G  A  R  L  A  E  Y  H  A  K  A  T  E  H  L  S  T  L  S  E 
aaagctaaaccggctctggaggacttgcgtcagggcttgcttccggtacttgaatcattc
 K  A  K  P  A  L  E  D  L  R  Q  G  L  L  P  V  L  E  S  F 
aaggtgtcctttctgtctgccttagaagagtatactaagaagcttaacacacaactgcct
 K  V  S  F  L  S  A  L  E  E  Y  T  K  K  L  N  T  Q  L  P 
ggtaccggggatcgggaggttcaggtgggcaagctaaacctcaaattccgaaagataaa
 G  T  G  G  S  G  G  S  G  G  Q  A  K  P  Q  I  P  K  D  K 
tcgaaagtggcaggctatattgaaattccagatgctgatattaaagaaccagtatatcca
 S  K  V  A  G  Y  I  E  I  P  D  A  D  I  K  E  P  V  Y  P 
ggaccagcaacacctgaacaattaaatagaggtgtaagctttgcagaagaaaatgaatca
 G  P  A  T  P  E  Q  L  N  R  G  V  S  F  A  E  E  N  E  S 
ctagatgatcaaaatatttcaattgcaggacacactttcattgaccgtccgaactatcaa
 L  D  D  Q  N  I  S  I  A  G  H  T  F  I  D  R  P  N  Y  Q 
tttacaaatcttaaagcagccaaaaaaggtagtatggtgtactttaaagttggtaatgaa
 F  T  N  L  K  A  A  K  K  G  S  M  V  Y  F  K  V  G  N  E 
acacgtaagtataaaatgacaagtataagagatgttaagcctacagatgtaggagttcta
 T  R  K  Y  K  M  T  S  I  R  D  V  K  P  T  D  V  G  V  L 
gatgaacaaaaaggtaaagataaacaattaacattaattacttgtgatgattacaatgaa
 D  E  Q  K  G  K  D  K  Q  L  T  L  I  T  C  D  D  Y  N  E 
aagacaggcgtttgggaaaaacgtaaaatctttgtagctacagaagtcaaactcgagcac
 K  T  G  V  W  E  K  R  K  I  F  V  A  T  E  V  K  L  E  H 
caccaccaccaccaccatcatcatcattga
 H  H  H  H  H  H  H  H  H  - 
```

*8. a*MSP9-L_{12} (MSP9-LPGTG-L12-SrtA-His_{10})

```
atggcttcgtccgagaatttgtacttccaaggatcgacgttttccaagttacgcgaacag
 M  A  S  S  E  N  L  Y  F  Q  G  S  T  F  S  K  L  R  E  Q
ttaggacccgtaacgcaggaattctgggacaaccttgagaaagagacggaaggccttcgc
 L  G  P  V  T  Q  E  F  W  D  N  L  E  K  E  T  E  G  L  R
caggagatgtcaaaagaccttgaggaagtgaaggctaaggtacaaccctatctggacgat
 Q  E  M  S  K  D  L  E  E  V  K  A  K  V  Q  P  Y  L  D  D
tttcaaaagaagtggcaagaagaaatggagttgtatcgtcaaaaagttgaacctttgggg
 F  Q  K  K  W  Q  E  E  M  E  L  Y  R  Q  K  V  E  P  L  G
gaggagatgcgtgatcgcgcccgcgcccacgtggatgcattgcgcacgcatttagctcca
 E  E  M  R  D  R  A  R  A  H  V  D  A  L  R  T  H  L  A  P
tatagtgatgagttgcgccagcgtttggccgcacgtttagaggctttgaaagagaatggc
 Y  S  D  E  L  R  Q  R  L  A  A  R  L  E  A  L  K  E  N  G
ggtgcccgtctggccgagtaccatgcaaaggcgacagaacatttgtccaccttgagcgag
 G  A  R  L  A  E  Y  H  A  K  A  T  E  H  L  S  T  L  S  E
aaagctaaaccggctctggaggacttgcgtcagggcttgcttccggtacttgaatcattc
 K  A  K  P  A  L  E  D  L  R  Q  G  L  L  P  V  L  E  S  F
aaggtgtcctttctgtctgccttagaagagtatactaagaagcttaacacacaactgcct
 K  V  S  F  L  S  A  L  E  E  Y  T  K  K  L  N  T  Q  L  P
ggcacaggtgctgcagctttagagggtaccctggtgccgcgcagccaagctaaacctcaa
 G  T  G  A  A  A  L  E  G  T  L  V  P  R  S  Q  A  K  P  Q
attccgaaagataaatcgaaagtggcaggctatattgaaattccagatgctgatattaaa
 I  P  K  D  K  S  K  V  A  G  Y  I  E  I  P  D  A  D  I  K
gaaccagtatatccaggaccagcaacacctgaacaattaaatagaggtgtaagctttgca
 E  P  V  Y  P  G  P  A  T  P  E  Q  L  N  R  G  V  S  F  A
gaagaaaatgaatcactagatgatcaaaatatttcaattgcaggacacactttcattgac
 E  E  N  E  S  L  D  D  Q  N  I  S  I  A  G  H  T  F  I  D
cgtccgaactatcaatttacaaatcttaaagcagccaaaaaaggtagtatggtgtacttt
 R  P  N  Y  Q  F  T  N  L  K  A  A  K  K  G  S  M  V  Y  F
aaagttggtaatgaaacacgtaagtataaaatgacaagtataagagatgttaagcctaca
 K  V  G  N  E  T  R  K  Y  K  M  T  S  I  R  D  V  K  P  T
gatgtaggagttctagatgaacaaaaaggtaaagataaacaattaacattaattacttgt
 D  V  G  V  L  D  E  Q  K  G  K  D  K  Q  L  T  L  I  T  C
gatgattacaatgaaaagacaggcgtttgggaaaaacgtaaaatctttgtagctacagaa
 D  D  Y  N  E  K  T  G  V  W  E  K  R  K  I  F  V  A  T  E
gtcaaactcgagcaccaccaccaccaccaccatcatcatcattga
 V  K  L  E  H  H  H  H  H  H  H  H  H  H  -
```

*9. a*MSP9-L_{14D} (MSP9-LPGTG-L14D-SrtA-His_{10})

```
atggcttcgtccgagaatttgtacttccaaggatcgacgttttccaagttacgcgaacag
 M  A  S  S  E  N  L  Y  F  Q  G  S  T  F  S  K  L  R  E  Q
ttaggacccgtaacgcaggaattctgggacaaccttgagaaagagacggaaggccttcgc
 L  G  P  V  T  Q  E  F  W  D  N  L  E  K  E  T  E  G  L  R
caggagatgtcaaaagaccttgaggaagtgaaggctaaggtacaaccctatctggacgat
 Q  E  M  S  K  D  L  E  E  V  K  A  K  V  Q  P  Y  L  D  D
tttcaaaagaagtggcaagaagaaatggagttgtatcgtcaaaaagttgaacctttgggg
 F  Q  K  K  W  Q  E  E  M  E  L  Y  R  Q  K  V  E  P  L  G
gaggagatgcgtgatcgcgcccgcgcccacgtggatgcattgcgcacgcatttagctcca
 E  E  M  R  D  R  A  R  A  H  V  D  A  L  R  T  H  L  A  P
tatagtgatgagttgcgccagcgtttggccgcacgtttagaggctttgaaagagaatggc
 Y  S  D  E  L  R  Q  R  L  A  A  R  L  E  A  L  K  E  N  G
ggtgcccgtctggccgagtaccatgcaaaggcgacagaacatttgtccaccttgagcgag
 G  A  R  L  A  E  Y  H  A  K  A  T  E  H  L  S  T  L  S  E
aaagctaaaccggctctggaggacttgcgtcagggcttgcttccggtacttgaatcattc
 K  A  K  P  A  L  E  D  L  R  Q  G  L  L  P  V  L  E  S  F
aaggtgtcctttctgtctgccttagaagagtatactaagaagcttaacacacaactgcct
 K  V  S  F  L  S  A  L  E  E  Y  T  K  K  L  N  T  Q  L  P
ggtaccggggatcgggaggttcaggtgggtccggtggtagtggtgggagtcaagctaaa
 G  T  G  G  S  G  G  S  G  G  S  G  G  S  G  G  S  Q  A  K
cctcaaattccgaaagataaatcgaaagtggcaggctatattgaaattccagatgctgat
 P  Q  I  P  K  D  K  S  K  V  A  G  Y  I  E  I  P  D  A  D
attaaagaaccagtatatccaggaccagcaacacctgaacaattaaatagaggtgtaagc
 I  K  E  P  V  Y  P  G  P  A  T  P  E  Q  L  N  R  G  V  S
tttgcagaagaaaatgaatcactagatgatcaaaatatttcaattgcaggacacactttc
 F  A  E  E  N  E  S  L  D  D  Q  N  I  S  I  A  G  H  T  F
attgaccgtccgaactatcaatttacaaatcttaaagcagccaaaaaaggtagtatggtg
 I  D  R  P  N  Y  Q  F  T  N  L  K  A  A  K  K  G  S  M  V
tactttaaagttggtaatgaaacacgtaagtataaaatgacaagtataagagatgttaag
 Y  F  K  V  G  N  E  T  R  K  Y  K  M  T  S  I  R  D  V  K
cctacagatgtaggagttctagatgaacaaaaaggtaaagataaacaattaacattaatt
 P  T  D  V  G  V  L  D  E  Q  K  G  K  D  K  Q  L  T  L  I
acttgtgatgattacaatgaaaagacaggcgtttgggaaaaacgtaaaatctttgtagct
 T  C  D  D  Y  N  E  K  T  G  V  W  E  K  R  K  I  F  V  A
acagaagtcaaactcgagcaccaccaccaccaccaccatcatcatcattga
 T  E  V  K  L  E  H  H  H  H  H  H  H  H  H  H  -
```

10. a-i-MSP9-L_{14D}-SrtA (Inhibitory-MSP9-LPGTG-L14D-SrtA-His_{10})

*Underlined is the inhibitory sequence at the N-terminal of the TEV cleavage site.

atggccagttct***ttacctcgtgacgcg***gaaaacctgtattttcagggatcgacgttttcc
M A S S **<u>L P R D A</u>** E N L Y F Q G S T F S
aagttacgcgaacagttaggacccgtaacgcaggaattctgggacaaccttgagaaagag
K L R E Q L G P V T Q E F W D N L E K E
acggaaggccttcgccaggagatgtcaaaagaccttgaggaagtgaaggctaaggtacaa
T E G L R Q E M S K D L E E V K A K V Q
ccctatctggacgattttcaaaagaagtggcaagaagaaatggagttgtatcgtcaaaaa
P Y L D D F Q K K W Q E E M E L Y R Q K
gttgaacctttgggggaggagatgcgtgatcgcgcccgcgcccacgtggatgcattgcgc
V E P L G E E M R D R A R A H V D A L R
acgcatttagctccatatagtgatgagttgcgccagcgtttggccgcacgtttagaggct
T H L A P Y S D E L R Q R L A A R L E A
ttgaaagagaatggcggtgcccgtctggccgagtaccatgcaaaggcgacagaacatttg
L K E N G G A R L A E Y H A K A T E H L
tccaccttgagcgagaaagctaaaccggctctggaggacttgcgtcagggcttgcttccg
S T L S E K A K P A L E D L R Q G L L P
gtacttgaatcattcaaggtgtcctttctgtctgccttagaagagtatactaagaagctt
V L E S F K V S F L S A L E E Y T K K L
aacacacaactgcctggtaccggggatcgggaggttcaggtgggtccggtggtagtggt
N T Q L P G T G G S G G S G G S G G S G
gggagtcaagctaaacctcaaattccgaaagataaatcgaaagtggcaggctatattgaa
G S Q A K P Q I P K D K S K V A G Y I E
attccagatgctgatattaaagaaccagtatatccaggaccagcaacacctgaacaatta
I P D A D I K E P V Y P G P A T P E Q L
aatagaggtgtaagctttgcagaagaaaatgaatcactagatgatcaaaatatttcaatt
N R G V S F A E E N E S L D D Q N I S I
gcaggacacactttcattgaccgtccgaactatcaatttacaaatcttaaagcagccaaa
A G H T F I D R P N Y Q F T N L K A A K
aaaggtagtatggtgtactttaaagttggtaatgaaacacgtaagtataaaatgacaagt
K G S M V Y F K V G N E T R K Y K M T S
ataagagatgttaagcctacagatgtaggagttctagatgaacaaaaaggtaaagataaa
I R D V K P T D V G V L D E Q K G K D K
caattaacattaattacttgtgatgattacaatgaaaagacaggcgtttgggaaaaacgt
Q L T L I T C D D Y N E K T G V W E K R
aaaatctttgtagctacagaagtcaaactcgagcaccaccaccaccaccaccatcatcat
K I F V A T E V K L E H H H H H H H H
cattga
H -

*11. a*MSP9-L$_{14D}$-eSrtA (MSP9-LPGTG-L14D-eSrtA-His$_{10}$)

```
atggcttcgtccgagaatttgtacttccaaggatcgacgttttccaagttacgcgaacag
 M  A  S  S  E  N  L  Y  F  Q  G  S  T  F  S  K  L  R  E  Q
ttaggacccgtaacgcaggaattctgggacaaccttgagaaagagacggaaggccttcgc
 L  G  P  V  T  Q  E  F  W  D  N  L  E  K  E  T  E  G  L  R
caggagatgtcaaaagaccttgaggaagtgaaggctaaggtacaaccctatctggacgat
 Q  E  M  S  K  D  L  E  E  V  K  A  K  V  Q  P  Y  L  D  D
tttcaaaagaagtggcaagaagaaatggagttgtatcgtcaaaaagttgaacctttgggg
 F  Q  K  K  W  Q  E  E  M  E  L  Y  R  Q  K  V  E  P  L  G
gaggagatgcgtgatcgcgcccgcgcccacgtggatgcattgcgcacgcatttagctcca
 E  E  M  R  D  R  A  R  A  H  V  D  A  L  R  T  H  L  A  P
tatagtgatgagttgcgccagcgtttggccgcacgtttagaggctttgaaagagaatggc
 Y  S  D  E  L  R  Q  R  L  A  A  R  L  E  A  L  K  E  N  G
ggtgcccgtctggccgagtaccatgcaaaggcgacagaacatttgtccaccttgagcgag
 G  A  R  L  A  E  Y  H  A  K  A  T  E  H  L  S  T  L  S  E
aaagctaaaccggctctggaggacttgcgtcagggcttgcttccggtacttgaatcattc
 K  A  K  P  A  L  E  D  L  R  Q  G  L  L  P  V  L  E  S  F
aaggtgtcctttctgtctgccttagaagagtatactaagaagcttaacacacaactgcct
 K  V  S  F  L  S  A  L  E  E  Y  T  K  K  L  N  T  Q  L  P
ggtaccgggggatcgggaggttcaggtgggtccggtggtagtggtgggagtcaagctaaa
 G  T  G  G  S  G  G  S  G  G  S  G  G  S  G  G  S  Q  A  K
ccgcagatccccaaagacaaatctaaagttgcaggttatattgagatcccagacgcggat
 P  Q  I  P  K  D  K  S  K  V  A  G  Y  I  E  I  P  D  A  D
attaaggagcccgtgtatccgggtcccgccactcgcgagcagttgaatcgcggagtctcc
 I  K  E  P  V  Y  P  G  P  A  T  R  E  Q  L  N  R  G  V  S
tttgcagaggaaaatgaatcgttggatgaccagaatatctctattgccggtcatacattc
 F  A  E  E  N  E  S  L  D  D  Q  N  I  S  I  A  G  H  T  F
atcgaccgtccaaattaccaattcactaaccttaaagccgcgaaaaaggggtcgatggtc
 I  D  R  P  N  Y  Q  F  T  N  L  K  A  A  K  K  G  S  M  V
tatttcaaggtgggcaatgaaacacgcaaatataaaatgacttcgattcgtaacgtcaaa
 Y  F  K  V  G  N  E  T  R  K  Y  K  M  T  S  I  R  N  V  K
ccaacggctgtggaagtgttagacgagcaaaaaggcaaggataagcaacttactttaatt
 P  T  A  V  E  V  L  D  E  Q  K  G  K  D  K  Q  L  T  L  I
acgtgtgacgattataatgaagagacaggagtatgggagacacgcaaaatcttcgtggcg
 T  C  D  D  Y  N  E  E  T  G  V  W  E  T  R  K  I  F  V  A
acggaggttaagctcgagcaccaccaccaccaccaccatcatcatcattga
 T  E  V  K  L  E  H  H  H  H  H  H  H  H  H  H  -
```

*12. a*MSP11-L_{12} (MSP11-LPGTG-L12-SrtA-His_{10})

```
atggctagcagcgaaaacctgtattttcagggcagcacctttagcaaactgcgtgaacag
 M  A  S  S  E  N  L  Y  F  Q  G  S  T  F  S  K  L  R  E  Q
ctgggcccggtgacccaggaattttgggataacctggaaaaagaaaccgaaggcctgcgt
 L  G  P  V  T  Q  E  F  W  D  N  L  E  K  E  T  E  G  L  R
caggaaatgagcaaagatctggaagaggtgaaagcgaaagtgcagccgtatctggatgac
 Q  E  M  S  K  D  L  E  E  V  K  A  K  V  Q  P  Y  L  D  D
tttcagaaaaaatggcaggaagagatggaactgtatcgtcagaaagtggaaccgctgcgt
 F  Q  K  K  W  Q  E  E  M  E  L  Y  R  Q  K  V  E  P  L  R
gcggaactgcaggaaggcgcgcgtcagaaactgcatgaactgcaggaaaaactgagcccg
 A  E  L  Q  E  G  A  R  Q  K  L  H  E  L  Q  E  K  L  S  P
ctgggcgaagagatgcgtgatcgtgcgcgtgcgcatgtggatgcgctgcgtacccatctg
 L  G  E  E  M  R  D  R  A  R  A  H  V  D  A  L  R  T  H  L
gcgccgtatagcgatgaactgcgtcagcgtctggcggcccgtctggaagcgctgaaagaa
 A  P  Y  S  D  E  L  R  Q  R  L  A  A  R  L  E  A  L  K  E
aacggcggtgcgcgtctggcggaatatcatgcgaaagcgaccgaacatctgagcaccctg
 N  G  G  A  R  L  A  E  Y  H  A  K  A  T  E  H  L  S  T  L
agcgaaaaagcgaaaccggcgctggaagatctgcgtcagggcctgctgccggtgctggaa
 S  E  K  A  K  P  A  L  E  D  L  R  Q  G  L  L  P  V  L  E
agctttaaagtgagctttctgagcgcgctggaagagtataccaaaaaactgaacacccag
 S  F  K  V  S  F  L  S  A  L  E  E  Y  T  K  K  L  N  T  Q
ctgccgggtacgggcgccgctgcactggaaggtaccctggtgccgcgcagccaagctaaa
 L  P  G  T  G  A  A  A  L  E  G  T  L  V  P  R  S  Q  A  K
cctcaaattccgaaagataaatcgaaagtggcaggctatattgaaattccagatgctgat
 P  Q  I  P  K  D  K  S  K  V  A  G  Y  I  E  I  P  D  A  D
attaaagaaccagtatatccaggaccagcaacacctgaacaattaaatagaggtgtaagc
 I  K  E  P  V  Y  P  G  P  A  T  P  E  Q  L  N  R  G  V  S
tttgcagaagaaaatgaatcactagatgatcaaaatatttcaattgcaggacacactttc
 F  A  E  E  N  E  S  L  D  D  Q  N  I  S  I  A  G  H  T  F
attgaccgtccgaactatcaatttacaaatcttaaagcagccaaaaaaggtagtatggtg
 I  D  R  P  N  Y  Q  F  T  N  L  K  A  A  K  K  G  S  M  V
tactttaaagttggtaatgaaacacgtaagtataaaatgacaagtataagagatgttaag
 Y  F  K  V  G  N  E  T  R  K  Y  K  M  T  S  I  R  D  V  K
cctacagatgtaggagttctagatgaacaaaaaggtaaagataaacaattaacattaatt
 P  T  D  V  G  V  L  D  E  Q  K  G  K  D  K  Q  L  T  L  I
acttgtgatgattacaatgaaaagacaggcgtttgggaaaaacgtaaaatctttgtagct
 T  C  D  D  Y  N  E  K  T  G  V  W  E  K  R  K  I  F  V  A
acagaagtcaaactcgagcaccaccaccaccaccaccatcatcatcattga
 T  E  V  K  L  E  H  H  H  H  H  H  H  H  H  H  -
```

_13. a_MSP11-L$_{14D}$ (MSP11-LPGTG-L14D-SrtA-His$_{10}$)

```
atggctagcagcgaaaacctgtattttcagggcagcacctttagcaaactgcgtgaacag
 M  A  S  S  E  N  L  Y  F  Q  G  S  T  F  S  K  L  R  E  Q
ctgggcccggtgacccaggaattttgggataacctggaaaaagaaaccgaaggcctgcgt
 L  G  P  V  T  Q  E  F  W  D  N  L  E  K  E  T  E  G  L  R
caggaaatgagcaaagatctggaagaggtgaaagcgaaagtgcagccgtatctggatgac
 Q  E  M  S  K  D  L  E  E  V  K  A  K  V  Q  P  Y  L  D  D
tttcagaaaaaatggcaggaagagatggaactgtatcgtcagaaagtggaaccgctgcgt
 F  Q  K  K  W  Q  E  E  M  E  L  Y  R  Q  K  V  E  P  L  R
gcggaactgcaggaaggcgcgcgtcagaaactgcatgaactgcaggaaaaactgagcccg
 A  E  L  Q  E  G  A  R  Q  K  L  H  E  L  Q  E  K  L  S  P
ctgggcgaagagatgcgtgatcgtgcgcgtgcgcatgtggatgcgctgcgtacccatctg
 L  G  E  E  M  R  D  R  A  R  A  H  V  D  A  L  R  T  H  L
gcgccgtatagcgatgaactgcgtcagcgtctggcggcccgtctggaagcgctgaaagaa
 A  P  Y  S  D  E  L  R  Q  R  L  A  A  R  L  E  A  L  K  E
aacggcggtgcgcgtctggcggaatatcatgcgaaagcgaccgaacatctgagcaccctg
 N  G  G  A  R  L  A  E  Y  H  A  K  A  T  E  H  L  S  T  L
agcgaaaaagcgaaaccggcgctggaagatctgcgtcagggcctgctgccggtgctggaa
 S  E  K  A  K  P  A  L  E  D  L  R  Q  G  L  L  P  V  L  E
agctttaaagtgagctttctgagcgcgctggaagagtataccaaaaaactgaacacccag
 S  F  K  V  S  F  L  S  A  L  E  E  Y  T  K  K  L  N  T  Q
ctgccgggtaccggggatcgggaggttcaggtgggtccggtggtagtggtgggagtcaa
 L  P  G  T  G  G  S  G  G  S  G  G  S  G  G  S  G  G  S  Q
gctaaacctcaaattccgaaagataaatcgaaagtggcaggctatattgaaattccagat
 A  K  P  Q  I  P  K  D  K  S  K  V  A  G  Y  I  E  I  P  D
gctgatattaaagaaccagtatatccaggaccagcaacacctgaacaattaaatagaggt
 A  D  I  K  E  P  V  Y  P  G  P  A  T  P  E  Q  L  N  R  G
gtaagctttgcagaagaaaatgaatcactagatgatcaaaatatttcaattgcaggacac
 V  S  F  A  E  E  N  E  S  L  D  D  Q  N  I  S  I  A  G  H
actttcattgaccgtccgaactatcaatttacaaatcttaaagcagccaaaaaaggtagt
 T  F  I  D  R  P  N  Y  Q  F  T  N  L  K  A  A  K  K  G  S
atggtgtactttaaagttggtaatgaaacacgtaagtataaaatgacaagtataagagat
 M  V  Y  F  K  V  G  N  E  T  R  K  Y  K  M  T  S  I  R  D
gttaagcctacagatgtaggagttctagatgaacaaaaaggtaaagataaacaattaaca
 V  K  P  T  D  V  G  V  L  D  E  Q  K  G  K  D  K  Q  L  T
ttaattacttgtgatgattacaatgaaaagacaggcgtttgggaaaaacgtaaaatcttt
 L  I  T  C  D  D  Y  N  E  K  T  G  V  W  E  K  R  K  I  F
gtagctacagaagtcaaactcgagcaccaccaccaccaccaccatcatcatcattga
 V  A  T  E  V  K  L  E  H  H  H  H  H  H  H  H  H  H  -
```

*14. a*MSP20-L_{12} (MSP20-LPGTG-L12-SrtA-His_{10})

```
atggccagttctgaaaacctgtattttcagggatcgacgttttccaagttacgtgagcag
 M   A   S   S   E   N   L   Y   F   Q   G   S   T   F   S   K   L   R   E   Q
ttaggacctgttacacaagagttctgggataacttagagaaagagacagaagggctgcgt
 L   G   P   V   T   Q   E   F   W   D   N   L   E   K   E   T   E   G   L   R
caagagatgagtaaagaccttgaagaagttaaagcaaaggttcagccctatctggatgat
 Q   E   M   S   K   D   L   E   E   V   K   A   K   V   Q   P   Y   L   D   D
ttccagaagaaatggcaggaggaaatggaattataccgtcagaaggtagagccacttcgt
 F   Q   K   K   W   Q   E   E   M   E   L   Y   R   Q   K   V   E   P   L   R
gcagaattgcaagaaggcgcacgccagaagttacacgaactgcaagaaaaattatcacct
 A   E   L   Q   E   G   A   R   Q   K   L   H   E   L   Q   E   K   L   S   P
ttaggggaggagatgcgcgaccgtgcacgcgcgcacgttgacgccttacgtacgcatctg
 L   G   E   E   M   R   D   R   A   R   A   H   V   D   A   L   R   T   H   L
gcgccgtactctgacgaattacgtcagcgcttagccgcgcgcttagaggccttaaaggag
 A   P   Y   S   D   E   L   R   Q   R   L   A   A   R   L   E   A   L   K   E
aacgggggagcgcgtcttgcagagtaccatgccaaagccacggaacatctgtccaccttg
 N   G   G   A   R   L   A   E   Y   H   A   K   A   T   E   H   L   S   T   L
agcgagaaggcgaagccagcactggaagacttacgccagggtttactgccagtccttgag
 S   E   K   A   K   P   A   L   E   D   L   R   Q   G   L   L   P   V   L   E
tcttttaaagtatcgtttctttctgcgcttgaggaatacacgaagaagttaaacactcag
 S   F   K   V   S   F   L   S   A   L   E   E   Y   T   K   K   L   N   T   Q
ggtactccagttacacaggagttttgggataatttagaaaaagagactgaagggcttcgc
 G   T   P   V   T   Q   E   F   W   D   N   L   E   K   E   T   E   G   L   R
caagagatgtcgaaggatttagaagaggtaaaggcgaaggtccaaccttacctggacgat
 Q   E   M   S   K   D   L   E   E   V   K   A   K   V   Q   P   Y   L   D   D
ttccagaagaagtggcaagaagaaatggagttataccgtcagaaagtcgaacctttacgt
 F   Q   K   K   W   Q   E   E   M   E   L   Y   R   Q   K   V   E   P   L   R
gccgaattacaagaaggagcacgccaaaaacttcatgagcttcaggagaagctgtccccc
 A   E   L   Q   E   G   A   R   Q   K   L   H   E   L   Q   E   K   L   S   P
cttggtgaagagatgcgcgaccgtgcgcgtgctcatgtagatgcattacgtacccacctt
 L   G   E   E   M   R   D   R   A   R   A   H   V   D   A   L   R   T   H   L
gccccctatagcgatgagttacgtcagcgtcttgccgcccgcctggaagctttaaaagag
 A   P   Y   S   D   E   L   R   Q   R   L   A   A   R   L   E   A   L   K   E
aatggcggtgctcgtttagcagagtatcacgccaaggccaccgaacatctttcaacttta
 N   G   G   A   R   L   A   E   Y   H   A   K   A   T   E   H   L   S   T   L
tctgagaaagccaaacctgcgttagaagacttacgtcaagggcttctgcctgtcttagag
 S   E   K   A   K   P   A   L   E   D   L   R   Q   G   L   L   P   V   L   E
tcgttcaaggtttcatttctgtcggcgcttgaagaatatactaaaaagttaaatacacag
 S   F   K   V   S   F   L   S   A   L   E   E   Y   T   K   K   L   N   T   Q
ttacctggtacaggtgctgcagctttagagggtaccctggtgccgcgcagccaagctaaa
 L   P   G   T   G   A   A   A   L   E   G   T   L   V   P   R   S   Q   A   K
cctcaaattccgaaagataaatcgaaagtggcaggctatattgaaattccagatgctgat
 P   Q   I   P   K   D   K   S   K   V   A   G   Y   I   E   I   P   D   A   D
attaaagaaccagtatatccaggaccagcaacacctgaacaattaaatagaggtgtaagc
 I   K   E   P   V   Y   P   G   P   A   T   P   E   Q   L   N   R   G   V   S
tttgcagaagaaaatgaatcactagatgatcaaaatatttcaattgcaggacacactttc
```

```
 F  A  E  E  N  E  S  L  D  D  Q  N  I  S  I  A  G  H  T  F
attgaccgtccgaactatcaatttacaaatcttaaagcagccaaaaaaggtagtatggtg
 I  D  R  P  N  Y  Q  F  T  N  L  K  A  A  K  K  G  S  M  V
tactttaaagttggtaatgaaacacgtaagtataaaatgacaagtataagagatgttaag
 Y  F  K  V  G  N  E  T  R  K  Y  K  M  T  S  I  R  D  V  K
cctacagatgtaggagttctagatgaacaaaaaggtaaagataaacaattaacattaatt
 P  T  D  V  G  V  L  D  E  Q  K  G  K  D  K  Q  L  T  L  I
acttgtgatgattacaatgaaaagacaggcgtttgggaaaaacgtaaaatctttgtagct
 T  C  D  D  Y  N  E  K  T  G  V  W  E  K  R  K  I  F  V  A
acagaagtcaaactcgagcaccaccaccaccaccaccatcatcatcattga
 T  E  V  K  L  E  H  H  H  H  H  H  H  H  H  H  -
```

*15. a*MSP20-L_{14D} (MSP20-LPGTG-L14D-SrtA-His_{10})

```
atggcatcgtcggagaacttgtatttccaaggctctactttctcgaagttgcgtgagcag
 M   A   S   S   E   N   L   Y   F   Q   G   S   T   F   S   K   L   R   E   Q
ttgggacctgtgacacaagagttctgggataatttagaaaaggagacagaagggctgcgt
 L   G   P   V   T   Q   E   F   W   D   N   L   E   K   E   T   E   G   L   R
caagagatgagtaaagaccttgaagaagttaaagcaaaggtgcagccctatctggatgat
 Q   E   M   S   K   D   L   E   E   V   K   A   K   V   Q   P   Y   L   D   D
ttccaaaaaaaatggcaagaagaaatggaattataccgtcagaaggtagagccacttcgt
 F   Q   K   K   W   Q   E   E   M   E   L   Y   R   Q   K   V   E   P   L   R
gcagaattgcaagaaggcgcacgccagaagttgcacgaactgcaagaaaaattgtcacct
 A   E   L   Q   E   G   A   R   Q   K   L   H   E   L   Q   E   K   L   S   P
ttgggggaggagatgcgcgaccgtgcacgcgcgcacgttgacgccttgcgtacgcatctg
 L   G   E   E   M   R   D   R   A   R   A   H   V   D   A   L   R   T   H   L
gcgccgtactctgacgaattacgtcagcgcttggccgcgcgcttagaggccttgaaggag
 A   P   Y   S   D   E   L   R   Q   R   L   A   A   R   L   E   A   L   K   E
aacgggggagcgcgtcttgcagagtaccatgccaaagccacggaacatctgtccaccttg
 N   G   G   A   R   L   A   E   Y   H   A   K   A   T   E   H   L   S   T   L
agcgagaaggcgaagccagcactggaagacttacgccagggtttgctgccagtccttgag
 S   E   K   A   K   P   A   L   E   D   L   R   Q   G   L   L   P   V   L   E
tcttttaaagtatcgtttctttctgcgcttgaggaatacacgaagaagttaaacactcag
 S   F   K   V   S   F   L   S   A   L   E   E   Y   T   K   K   L   N   T   Q
ggtactccagtgacacaggagttttgggataatttggaaaaagagactgaagggcttcgc
 G   T   P   V   T   Q   E   F   W   D   N   L   E   K   E   T   E   G   L   R
caagagatgtcgaaggatttggaagaggtaaaggcgaaggtccaaccttacctggacgat
 Q   E   M   S   K   D   L   E   E   V   K   A   K   V   Q   P   Y   L   D   D
ttccaaaagaagtggcaggaagaaatggagttataccgtcagaaagtcgaacctttacgt
 F   Q   K   K   W   Q   E   E   M   E   L   Y   R   Q   K   V   E   P   L   R
gccgaattacaagaaggagcacgccaaaaacttcatgagcttcaggagaagctgtccccc
 A   E   L   Q   E   G   A   R   Q   K   L   H   E   L   Q   E   K   L   S   P
cttggtgaggagatgcgcgaccgtgcgcgtgctcatgtagatgcattacgtacccacctt
 L   G   E   E   M   R   D   R   A   R   A   H   V   D   A   L   R   T   H   L
gcccccctatagcgatgagttgcgtcagcgtcttgccgcccgcctggaagctttgaaagag
 A   P   Y   S   D   E   L   R   Q   R   L   A   A   R   L   E   A   L   K   E
aatggcggtgctcgtttagcagagtatcacgccaaggccaccgaacatctttcaactttg
 N   G   G   A   R   L   A   E   Y   H   A   K   A   T   E   H   L   S   T   L
tctgagaaagccaaacctgcgttagaagacttgcgtcaagggcttctgcctgtcttagag
 S   E   K   A   K   P   A   L   E   D   L   R   Q   G   L   L   P   V   L   E
tcgttcaaggtgtcatttctgtcggcgcttgaagaatatactaaaaagttgaatacacag
 S   F   K   V   S   F   L   S   A   L   E   E   Y   T   K   K   L   N   T   Q
ttacctggtaccggggatcgggaggttcaggtgggtccggtggtagtggtgggagtcaa
 L   P   G   T   G   G   S   G   G   S   G   G   S   G   G   S   G   G   S   Q
gctaaacctcaaattccgaaagataaatcgaaagtggcaggctatattgaaattccagat
 A   K   P   Q   I   P   K   D   K   S   K   V   A   G   Y   I   E   I   P   D
gctgatattaaagaaccagtatatccaggaccagcaacacctgaacaattaaatagaggt
 A   D   I   K   E   P   V   Y   P   G   P   A   T   P   E   Q   L   N   R   G
gtaagctttgcagaagaaaatgaatcactagatgatcaaaatatttcaattgcaggacac
```

```
 V  S  F  A  E  E  N  E  S  L  D  D  Q  N  I  S  I  A  G  H
actttcattgaccgtccgaactatcaatttacaaatcttaaagcagccaaaaaaggtagt
 T  F  I  D  R  P  N  Y  Q  F  T  N  L  K  A  A  K  K  G  S
atggtgtactttaaagttggtaatgaaacacgtaagtataaaatgacaagtataagagat
 M  V  Y  F  K  V  G  N  E  T  R  K  Y  K  M  T  S  I  R  D
gttaagcctacagatgtaggagttctagatgaacaaaaaggtaaagataaacaattaaca
 V  K  P  T  D  V  G  V  L  D  E  Q  K  G  K  D  K  Q  L  T
ttaattacttgtgatgattacaatgaaaagacaggcgtttgggaaaaacgtaaaatcttt
 L  I  T  C  D  D  Y  N  E  K  T  G  V  W  E  K  R  K  I  F
gtagctacagaagtcaaactcgagcaccaccaccaccaccaccatcatcatcattga
 V  A  T  E  V  K  L  E  H  H  H  H  H  H  H  H  H  H  -
```

16. a**SFTI-L$_{12}$ (SFTI-LPGTG-L12-SrtA-His$_{10}$)**

```
atggcttcgtccgagaatttgtacttccaaggacgctgcaccaaaagcattccgccgatt
 M  A  S  S  E  N  L  Y  F  Q  G  R  C  T  K  S  I  P  P  I
tgctttccggatctgcctggcacaggtgctgcagctttagagggtaccctggtgccgcgc
 C  F  P  D  L  P  G  T  G  A  A  A  L  E  G  T  L  V  P  R
agccaagctaaacctcaaattccgaaagataaatcgaaagtggcaggctatattgaaatt
 S  Q  A  K  P  Q  I  P  K  D  K  S  K  V  A  G  Y  I  E  I
ccagatgctgatattaaagaaccagtatatccaggaccagcaacacctgaacaattaaat
 P  D  A  D  I  K  E  P  V  Y  P  G  P  A  T  P  E  Q  L  N
agaggtgtaagctttgcagaagaaaatgaatcactagatgatcaaaatatttcaattgca
 R  G  V  S  F  A  E  E  N  E  S  L  D  D  Q  N  I  S  I  A
ggacacactttcattgaccgtccgaactatcaatttacaaatcttaaagcagccaaaaaa
 G  H  T  F  I  D  R  P  N  Y  Q  F  T  N  L  K  A  A  K  K
ggtagtatggtgtactttaaagttggtaatgaaacacgtaagtataaaatgacaagtata
 G  S  M  V  Y  F  K  V  G  N  E  T  R  K  Y  K  M  T  S  I
agagatgttaagcctacagatgtaggagttctagatgaacaaaaaggtaaagataaacaa
 R  D  V  K  P  T  D  V  G  V  L  D  E  Q  K  G  K  D  K  Q
ttaacattaattacttgtgatgattacaatgaaaagacaggcgtttgggaaaaacgtaaa
 L  T  L  I  T  C  D  D  Y  N  E  K  T  G  V  W  E  K  R  K
atctttgtagctacagaagtcaaactcgagcaccaccaccaccaccaccatcatcatcat
 I  F  V  A  T  E  V  K  L  E  H  H  H  H  H  H  H  H  H  H
tga
 -
```

*17. a*SFTI-L_{19D} (SFTI-LPGTG-L19D-SrtA-His_{10})

```
atggccagttctttacctcgtgacgcggaaaacctgtattttcagggacgctgcaccaaa
 M  A  S  S  L  P  R  D  A  E  N  L  Y  F  Q  G  R  C  T  K
agcattccgccgatttgctttccggatctgcctggtaccggggatcggaggttcaggt
 S  I  P  P  I  C  F  P  D  L  P  G  T  G  G  S  G  G  S  G
gggtccggtggtagtggtgggagtctcgtgccgcgctcccaagctaaacctcaaattccg
 G  S  G  G  S  G  G  S  L  V  P  R  S  Q  A  K  P  Q  I  P
aaagataaatcgaaagtggcaggctatattgaaattccagatgctgatattaaagaacca
 K  D  K  S  K  V  A  G  Y  I  E  I  P  D  A  D  I  K  E  P
gtatatccaggaccagcaacacctgaacaattaaatagaggtgtaagctttgcagaagaa
 V  Y  P  G  P  A  T  P  E  Q  L  N  R  G  V  S  F  A  E  E
aatgaatcactagatgatcaaaatatttcaattgcaggacacactttcattgaccgtccg
 N  E  S  L  D  D  Q  N  I  S  I  A  G  H  T  F  I  D  R  P
aactatcaatttacaaatcttaaagcagccaaaaaaggtagtatggtgtactttaaagtt
 N  Y  Q  F  T  N  L  K  A  A  K  K  G  S  M  V  Y  F  K  V
ggtaatgaaacacgtaagtataaaatgacaagtataagagatgttaagcctacagatgta
 G  N  E  T  R  K  Y  K  M  T  S  I  R  D  V  K  P  T  D  V
ggagttctagatgaacaaaaaggtaaagataaacaattaacattaattacttgtgatgat
 G  V  L  D  E  Q  K  G  K  D  K  Q  L  T  L  I  T  C  D  D
tacaatgaaaagacaggcgtttgggaaaaacgtaaaatctttgtagctacagaagtcaaa
 Y  N  E  K  T  G  V  W  E  K  R  K  I  F  V  A  T  E  V  K
ctcgagcaccaccaccaccaccaccatcatcatcattga
 L  E  H  H  H  H  H  H  H  H  H  H  -
```

*18. a*kB1-L_{12} (kB1-LPVTG-L12-SrtA-His_{10})

```
atggcttcgtccgagaatttgtacttccaaggatgcggcgaaacctgcgtgggcggcacc
 M  A  S  S  E  N  L  Y  F  Q  G  C  G  E  T  C  V  G  G  T
tgcaacaccccgggctgcacctgcagctggccggtgtgcacccgcaacggcctgcctgtg
 C  N  T  P  G  C  T  C  S  W  P  V  C  T  R  N  G  L  P  V
acaggtgctgcagctttagagggtaccctggtgccgcgcagccaagctaaacctcaaatt
 T  G  A  A  A  L  E  G  T  L  V  P  R  S  Q  A  K  P  Q  I
ccgaaagataaatcgaaagtggcaggctatattgaaattccagatgctgatattaaagaa
 P  K  D  K  S  K  V  A  G  Y  I  E  I  P  D  A  D  I  K  E
ccagtatatccaggaccagcaacacctgaacaattaaatagaggtgtaagctttgcagaa
 P  V  Y  P  G  P  A  T  P  E  Q  L  N  R  G  V  S  F  A  E
gaaaatgaatcactagatgatcaaaatatttcaattgcaggacacactttcattgaccgt
 E  N  E  S  L  D  D  Q  N  I  S  I  A  G  H  T  F  I  D  R
ccgaactatcaatttacaaatcttaaagcagccaaaaaaggtagtatggtgtactttaaa
 P  N  Y  Q  F  T  N  L  K  A  A  K  K  G  S  M  V  Y  F  K
gttggtaatgaaacacgtaagtataaaatgacaagtataagagatgttaagcctacagat
 V  G  N  E  T  R  K  Y  K  M  T  S  I  R  D  V  K  P  T  D
gtaggagttctagatgaacaaaaaggtaaagataaacaattaacattaattacttgtgat
 V  G  V  L  D  E  Q  K  G  K  D  K  Q  L  T  L  I  T  C  D
gattacaatgaaaagacaggcgtttgggaaaaacgtaaaatctttgtagctacagaagtc
 D  Y  N  E  K  T  G  V  W  E  K  R  K  I  F  V  A  T  E  V
aaactcgagcaccaccaccaccaccaccatcatcatcattga
 K  L  E  H  H  H  H  H  H  H  H  H  H  -
```

***19. a*kB1-L$_{19D}$ (kB1-LPVTG-L19D-SrtA-His$_{10}$)**

```
atggccagttctttacctcgtgacgcggaaaacctgtattttcagggatgcggcgaaacc
 M  A  S  S  L  P  R  D  A  E  N  L  Y  F  Q  G  C  G  E  T
tgcgtgggcggcacctgcaacaccccgggctgcacctgcagctggccggtgtgcacccgc
 C  V  G  G  T  C  N  T  P  G  C  T  C  S  W  P  V  C  T  R
aacggcctgccggtgaccgggggatcgggaggttcaggtgggtccggtggtagtggtggg
 N  G  L  P  V  T  G  G  S  G  G  S  G  G  S  G  G  S  G  G
agtctcgtgccgcgctcccaagctaaacctcaaattccgaaagataaatcgaaagtggca
 S  L  V  P  R  S  Q  A  K  P  Q  I  P  K  D  K  S  K  V  A
ggctatattgaaattccagatgctgatattaaagaaccagtatatccaggaccagcaaca
 G  Y  I  E  I  P  D  A  D  I  K  E  P  V  Y  P  G  P  A  T
cctgaacaattaaatagaggtgtaagctttgcagaagaaaatgaatcactagatgatcaa
 P  E  Q  L  N  R  G  V  S  F  A  E  E  N  E  S  L  D  D  Q
aatatttcaattgcaggacacactttcattgaccgtccgaactatcaatttacaaatctt
 N  I  S  I  A  G  H  T  F  I  D  R  P  N  Y  Q  F  T  N  L
aaagcagccaaaaaaggtagtatggtgtactttaaagttggtaatgaaacacgtaagtat
 K  A  A  K  K  G  S  M  V  Y  F  K  V  G  N  E  T  R  K  Y
aaaatgacaagtataagagatgttaagcctacagatgtaggagttctagatgaacaaaaa
 K  M  T  S  I  R  D  V  K  P  T  D  V  G  V  L  D  E  Q  K
ggtaaagataaacaattaacattaattacttgtgatgattacaatgaaaagacaggcgtt
 G  K  D  K  Q  L  T  L  I  T  C  D  D  Y  N  E  K  T  G  V
tgggaaaaacgtaaaatctttgtagctacagaagtcaaactcgagcaccaccaccaccac
 W  E  K  R  K  I  F  V  A  T  E  V  K  L  E  H  H  H  H  H
caccatcatcatcattga
 H  H  H  H  H  -
```

*20. a*Vc1.1-L_{12} (Vc1.1-LPGTG-L12-SrtA-His_{10})

```
atggcttcgtccgagaatttgtacttccaaggaggttgctgcagcgatccgcgctgcaac
 M  A  S  S  E  N  L  Y  F  Q  G  G  C  C  S  D  P  R  C  N
tatgatcatccggaaatttgcggtctgcctggcacaggtgctgcagctttagagggtacc
 Y  D  H  P  E  I  C  G  L  P  G  T  G  A  A  A  L  E  G  T
ctggtgccgcgcagccaagctaaacctcaaattccgaaagataaatcgaaagtggcaggc
 L  V  P  R  S  Q  A  K  P  Q  I  P  K  D  K  S  K  V  A  G
tatattgaaattccagatgctgatattaaagaaccagtatatccaggaccagcaacacct
 Y  I  E  I  P  D  A  D  I  K  E  P  V  Y  P  G  P  A  T  P
gaacaattaaatagaggtgtaagctttgcagaagaaaatgaatcactagatgatcaaaat
 E  Q  L  N  R  G  V  S  F  A  E  E  N  E  S  L  D  D  Q  N
atttcaattgcaggacacactttcattgaccgtccgaactatcaatttacaaatcttaaa
 I  S  I  A  G  H  T  F  I  D  R  P  N  Y  Q  F  T  N  L  K
gcagccaaaaaaggtagtatggtgtactttaaagttggtaatgaaacacgtaagtataaa
 A  A  K  K  G  S  M  V  Y  F  K  V  G  N  E  T  R  K  Y  K
atgacaagtataagagatgttaagcctacagatgtaggagttctagatgaacaaaaaggt
 M  T  S  I  R  D  V  K  P  T  D  V  G  V  L  D  E  Q  K  G
aaagataaacaattaacattaattacttgtgatgattacaatgaaaagacaggcgtttgg
 K  D  K  Q  L  T  L  I  T  C  D  D  Y  N  E  K  T  G  V  W
gaaaaacgtaaaatctttgtagctacagaagtcaaactcgagcaccaccaccaccaccac
 E  K  R  K  I  F  V  A  T  E  V  K  L  E  H  H  H  H  H  H
catcatcatcattga
 H  H  H  H  -
```

***21. a*Vc1.1-L_{19D} (Vc1.1-LPGTG-L19D-SrtA-His_{10})**

```
atggcttcgtccgagaatttgtacttccaaggaggttgctgcagcgatccgcgctgcaac
 M  A  S  S  E  N  L  Y  F  Q  G  G  C  C  S  D  P  R  C  N
tatgatcatccggaaatttgcggtctgcctggtaccggggatcgggaggttcaggtggg
 Y  D  H  P  E  I  C  G  L  P  G  T  G  G  S  G  G  S  G  G
tccggtggtagtggtgggagtctggtgccgcgcagccaagctaaacctcaaattccgaaa
 S  G  G  S  G  G  S  L  V  P  R  S  Q  A  K  P  Q  I  P  K
gataaatcgaaagtggcaggctatattgaaattccagatgctgatattaaagaaccagta
 D  K  S  K  V  A  G  Y  I  E  I  P  D  A  D  I  K  E  P  V
tatccaggaccagcaacacctgaacaattaaatagaggtgtaagctttgcagaagaaaat
 Y  P  G  P  A  T  P  E  Q  L  N  R  G  V  S  F  A  E  E  N
gaatcactagatgatcaaaatatttcaattgcaggacacactttcattgaccgtccgaac
 E  S  L  D  D  Q  N  I  S  I  A  G  H  T  F  I  D  R  P  N
tatcaatttacaaatcttaaagcagccaaaaaaggtagtatggtgtactttaaagttggt
 Y  Q  F  T  N  L  K  A  A  K  K  G  S  M  V  Y  F  K  V  G
aatgaaacacgtaagtataaaatgacaagtataagagatgttaagcctacagatgtagga
 N  E  T  R  K  Y  K  M  T  S  I  R  D  V  K  P  T  D  V  G
gttctagatgaacaaaaaggtaaagataaacaattaacattaattacttgtgatgattac
 V  L  D  E  Q  K  G  K  D  K  Q  L  T  L  I  T  C  D  D  Y
aatgaaaagacaggcgtttgggaaaaacgtaaaatctttgtagctacagaagtcaaactc
 N  E  K  T  G  V  W  E  K  R  K  I  F  V  A  T  E  V  K  L
gagcaccaccaccaccaccaccatcatcatcattga
 E  H  H  H  H  H  H  H  H  H  H  -
```
