# Supplementary material for: Self-cyclisation as a general and efficient platform for peptide and protein macrocyclisation
Source: Commun Chem. 2023 Mar 4;6:48. doi: 10.1038/s42004-023-00841-5 (PMC9985607; doi:10.1038/s42004-023-00841-5)
Supplement: Supplementary file 7 — Reporting Summary [file 42004_2023_841_MOESM7_ESM.pdf]

Corresponding author(s): Mehdi Mobli
Last updated by author(s): Feb 9, 2023

Reporting Summary

Nature Portfolio wishes to improve the reproducibility of the work that we publish. This form provides structure for consistency and transparency in reporting. For further information on Nature Portfolio policies, see our Editorial Policies and the Editorial Policy Checklist.

Statistics

For all statistical analyses, confirm that the following items are present in the figure legend, table legend, main text, or Methods section.

n/a	Confirmed	
☐	☒	The exact sample size (*n*) for each experimental group/condition, given as a discrete number and unit of measurement
☐	☒	A statement on whether measurements were taken from distinct samples or whether the same sample was measured repeatedly
☐	☒	The statistical test(s) used AND whether they are one- or two-sided<br>*Only common tests should be described solely by name; describe more complex techniques in the Methods section.*
☒	☐	A description of all covariates tested
☒	☐	A description of any assumptions or corrections, such as tests of normality and adjustment for multiple comparisons
☐	☒	A full description of the statistical parameters including central tendency (e.g. means) or other basic estimates (e.g. regression coefficient) AND variation (e.g. standard deviation) or associated estimates of uncertainty (e.g. confidence intervals)
☐	☒	For null hypothesis testing, the test statistic (e.g. *F*, *t*, *r*) with confidence intervals, effect sizes, degrees of freedom and *P* value noted<br>*Give P values as exact values whenever suitable.*
☒	☐	For Bayesian analysis, information on the choice of priors and Markov chain Monte Carlo settings
☒	☐	For hierarchical and complex designs, identification of the appropriate level for tests and full reporting of outcomes
☒	☐	Estimates of effect sizes (e.g. Cohen's *d*, Pearson's *r*), indicating how they were calculated

Our web collection on statistics for biologists contains articles on many of the points above.

Software and code

Policy information about availability of computer code

Data collection	- The NMR data were acquired on a Bruker NMR spectrometer using the Topspin v4.1.3 software (available from Bruker Biospin - https://www.bruker.com/en.html).
Data analysis	- The NMR data were processed using either Topspin Topspin v4.1.3 or the Rowland NMR toolkit V3 (available from https://rnmrtk.uchc.edu/).<br>- The NMR data were analysed using ccpnmr (available from https://ccpn.ac.uk/) version v2.4.1.<br>- GraphPad Prism v8.3.1 and Microsoft Excel (Office 365) used to produce plots and calculate statistics (http://www.graphpad.com)<br>- Animal images were analysed using Living Image Software V4.7.4 (Perkin Elmer).<br>- Molecular dynamics performed using GROMOS11 software package.<br>- Bruker Compass QuantAnalysis V2.1 used to for analysis of MS data.<br>- Band intensity of Coomassie staining was quantified using ImageLab software V5.2 build 14 (Bio-Rad).

For manuscripts utilizing custom algorithms or software that are central to the research but not yet described in published literature, software must be made available to editors and reviewers. We strongly encourage code deposition in a community repository (e.g. GitHub). See the Nature Portfolio guidelines for submitting code & software for further information.

Data

Policy information about availability of data

All manuscripts must include a data availability statement. This statement should provide the following information, where applicable:

- Accession codes, unique identifiers, or web links for publicly available datasets
- A description of any restrictions on data availability
- For clinical datasets or third party data, please ensure that the statement adheres to our policy

The data that support the findings of this study are available in the supplementary sections or from the corresponding author upon reasonable request.

Human research participants

Policy information about studies involving human research participants and Sex and Gender in Research.

Reporting on sex and gender	*Use the terms sex (biological attribute) and gender (shaped by social and cultural circumstances) carefully in order to avoid confusing both terms. Indicate if findings apply to only one sex or gender; describe whether sex and gender were considered in study design whether sex and/or gender was determined based on self-reporting or assigned and methods used. Provide in the source data disaggregated sex and gender data where this information has been collected, and consent has been obtained for sharing of individual-level data; provide overall numbers in this Reporting Summary. Please state if this information has not been collected. Report sex- and gender-based analyses where performed, justify reasons for lack of sex- and gender-based analysis.*
Population characteristics	*Describe the covariate-relevant population characteristics of the human research participants (e.g. age, genotypic information, past and current diagnosis and treatment categories). If you filled out the behavioural & social sciences study design questions and have nothing to add here, write "See above."*
Recruitment	*Describe how participants were recruited. Outline any potential self-selection bias or other biases that may be present and how these are likely to impact results.*
Ethics oversight	*Identify the organization(s) that approved the study protocol.*

Note that full information on the approval of the study protocol must also be provided in the manuscript.

Field-specific reporting

Please select the one below that is the best fit for your research. If you are not sure, read the appropriate sections before making your selection.

☒ Life sciences ☐ Behavioural & social sciences ☐ Ecological, evolutionary & environmental sciences

For a reference copy of the document with all sections, see nature.com/documents/nr-reporting-summary-flat.pdf

Life sciences study design

All studies must disclose on these points even when the disclosure is negative.

Sample size	Sample size is reported where relevant (i.e. number of animals, replicates etc.).
Data exclusions	No data was excluded.
Replication	At least three replicates were generated for the kinetics (mass spectrometry), protein yields and imaging experiments from which statistical data were derived. All attempts at replication were successful other than that described under Data exclusions.
Randomization	No randomisation was necessary as absolute measurements were determined by computer software.
Blinding	No blinding was required as the data did not involve experiments that are influenced by observers.

Reporting for specific materials, systems and methods

We require information from authors about some types of materials, experimental systems and methods used in many studies. Here, indicate whether each material, system or method listed is relevant to your study. If you are not sure if a list item applies to your research, read the appropriate section before selecting a response.

Materials & experimental systems

n/a	Involved in the study
☒	☐ Antibodies
☒	☐ Eukaryotic cell lines
☒	☐ Palaeontology and archaeology
☐	☒ Animals and other organisms
☒	☐ Clinical data
☒	☐ Dual use research of concern

Methods

n/a	Involved in the study
☒	☐ ChIP-seq
☒	☐ Flow cytometry
☒	☐ MRI-based neuroimaging

Animals and other research organisms

Policy information about studies involving animals; ARRIVE guidelines recommended for reporting animal research, and Sex and Gender in Research

Laboratory animals	Naïve female BALB/c nude mice 2-16 months.
Wild animals	The study did not involve wild animals.
Reporting on sex	Only female mice were used, no sex-based analysis was performed - not required to support the claims of the manuscript.
Field-collected samples	No samples were collected from the field.
Ethics oversight	This study was carried out in accordance with the recommendations of Animal Care and Protection Act (2001) and the Australian code for the care and use of animals for scientific purposes (8th Edition). The protocol was approved by a University of Queensland Anatomical Bioscience Ethics Committee (Approval AIBN/105/19).

Note that full information on the approval of the study protocol must also be provided in the manuscript.
